# Comparison between Standard and High-Definition Multi-Electrode Mapping Catheter in Ventricular Tachycardia Ablation

**DOI:** 10.3390/jcdd9080232

**Published:** 2022-07-22

**Authors:** Sergio Conti, Francesco Sabatino, Gabriele De Blasi, Giuseppe Di Stabile, Giuseppe Sgarito

**Affiliations:** ARNAS Civico Hospital, 90127 Palermo, Italy; francesco1848@gmail.com (F.S.); gabrieledeblasiabc@gmail.com (G.D.B.); giuseppedistabile5@gmail.com (G.D.S.); giuseppe.sgarito@gmail.com (G.S.)

**Keywords:** ventricular tachycardia, catheter ablation, mapping, multipolar mapping, late potentials, local abnormal ventricular activities

## Abstract

A high-definition mapping catheter has been introduced, allowing for bipolar recording along and across the spline with a rapid assessment of voltage, activation, and directionality of conduction. We aimed to evaluate differences in mapping density, accuracy, time, and consequently RF time between different mapping catheters used for ventricular tachycardia (VT) ablation. We enrolled consecutive patients undergoing VT ablation at our center. Patients were divided into the LiveWire 2-2-2 mm catheter (group A) and the HD Grid SE (group B). Primary endpoints were total RF delivery time, the number of points acquired in sinus rhythm and VT, and the scar area. Fifty-one patients were enrolled, 22 in group A and 29 in group B. More points were acquired in the Grid group in sinus rhythm (SR) and during VT (2060.78 ± 1600.38 vs. 3278.63 ± 3214.45, *p* = 0.05; 4201.13 ± 5141.61 vs. 10,569.43 ± 13,644.94, *p* = 0.02, respectively). The scar area was smaller in group B (Bipolar area, cm^2^ 4.52 ± 2.72 vs. 2.89 ± 2.81, *p* = 0.05. Unipolar area, cm^2^ 7.47 ± 4.55 vs. 5.56 ± 2.79, *p* = 0.03). Radiofrequency (RF) time was shorter in the Grid group (30.52 ± 13.94 vs. 22.16 ± 11.03, *p* = 0.014). LPs and LAVAs were eliminated in overall >93% of patients. No differences were found in terms of arrhythmia-free survival at follow-up. In conclusion, the use of a high-definition mapping catheter was associated with significantly shorter mapping time during VT and RF time. Significantly more points were acquired in SR and during VT. During remap, we also observed more LAVAs and LPs requiring further ablation.

## 1. Introduction

**Figure 1:**
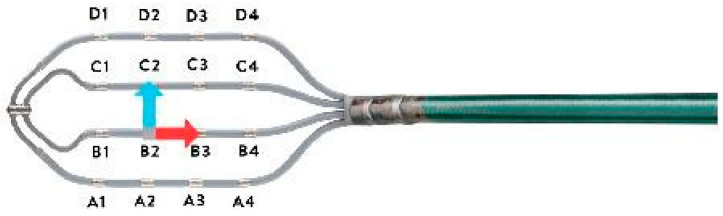
The Advisor^TM^ HD Grid mapping catheter SE (Abbott Medical, MN, USA) has a rectangular shape consisting of 16 electrodes distributed across four splines (4 electrodes [3 mm electrode] per spline with an interelectrode distance of 3 mm), allowing for bipolar recording along and across the splines.

High-definition mapping is becoming the cornerstone of complex atrial and ventricular arrhythmia ablation procedures [1,2]. The Advisor^TM^ HD Grid mapping catheter SE (Abbott Medical, MN, USA) is widely used to create high-definition substrate and activation maps. It has a rectangular shape consisting of 16 electrodes distributed across four splines (4 electrodes [3 mm electrode] per spline with an interelectrode distance of 3 mm), allowing for bipolar recording along and across the splines (Figure 1). In combination with the automated HD wave algorithm, the Grid catheter rapidly assesses voltage, activation, and directionality of conduction, creating accurate high-resolution maps. It is essential to stress the concept of the wavefront direction in relation to the recording mapping catheter’s orientation. A wavefront propagating perpendicular to the recording bipole axis produces no difference in potential between the electrodes, hence no signal [3,4]. Theoretically, it would seem that this could significantly impact “voltage maps” that display areas of infarction and scar as low-amplitude regions. The impact of recording bipole orientation on electrogram amplitude may be reduced by nonuniform, anisotropic conduction, particularly in low-voltage areas. The Advisor HD Grid Catheter SE acquiring simultaneous signals across orthogonal planes may adequately assess the EGM voltage unrelated to the activation front’s directionality. Tung et al. examined the effect of the activation direction on the voltage map [5]. They demonstrated the impact of different wavefronts on the voltage by pacing from various ventricular sites during substrate-based VT ablation. Overlap of the scar area on the map between different wavefronts was analyzed. A different activation front revealed 22% variability in a median bipolar defined scar area (<0.5 mV), and the concordance between wavefronts was lower in patients with mixed scar (0.5–1.5 mV) compared to those with dense scar (<0.5 mV). We aimed to evaluate how the Advisor HD Grid SE mapping catheter impacts mapping density, accuracy, timing, and acute outcomes compared to a standard linear mapping catheter widely used for VT mapping.

## 2. Materials and Methods

We prospectively included fifty-one consecutive patients undergoing VT ablation in this study at our Centre. Patients were divided into two different groups according to the mapping catheter used: the standard linear LiveWire 2-2-2 mm (Abbott Medical) mapping catheter (group A) and the HD Grid SE (Abbott Medical) mapping catheter (group B). Acute outcomes were defined as LPs and LAVAs complete elimination and acute VT inducibility.

### 2.1. Inclusion Criteria

- Patient aged 18 years and over;
- Patients affected by VT (ICM, NICM, myocarditis);
- Patients who undergo ablation with support of EnSite Precision Electroanatomical Mapping System (Abbott Medical) and high-density mapping catheter LiveWire and Advisor HD Grid SE;
- Patient having signed an Informed Consent.

### 2.2. Exclusion Criteria

- Patient unwilling or unable to consent;
- Presence of any contraindications to VT ablation;
- Pregnancy or breastfeeding;
- Comorbidities with life expectancy < 1 year.

### 2.3. Primary and Secondary Endpoints

The study’s primary endpoints were the total RF delivery time, the number of points acquired in sinus rhythm and VT, and the scar area. These endpoints were chosen because we hypothesized that HD Grid could provide a much more detailed map with more acquired and used points and consequently less RF time. In addition, complication rates also mirror RF time, so the endpoint also reflects safety. Secondary endpoints included procedural time, percentage of LAVAs and LPs abolition, and arrhythmia recurrence during follow-up.

### 2.4. Mapping, Induction, and Ablation Procedure

**Figure 2:**
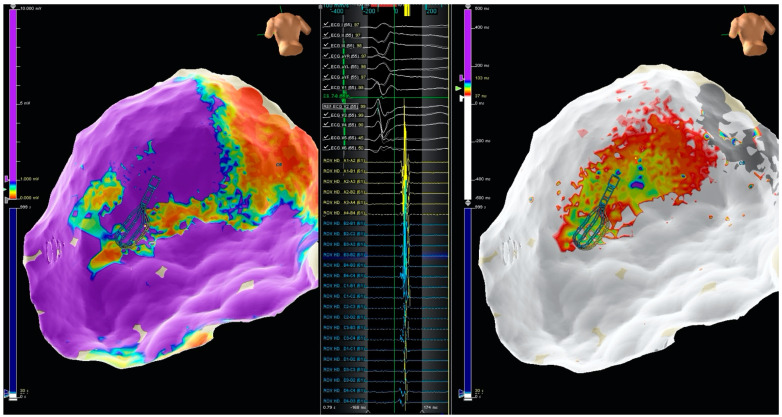
On the left panel an epicardial substrate map. In the middle panel, LPs mapped using the HD Grid multipolar mapping catheter. In the right panel activation map of the LPs area.

Procedures were performed after obtaining informed consent from all patients. All procedures were performed under local anesthesia and conscious sedation. General anesthesia was used in selected patients, particularly when epicardial access was previously planned. Antiarrhythmic drugs were discontinued at least four weeks before the procedure unless the patients were admitted for VT storm. Hemodynamic parameters were continuously monitored, such as invasive blood pressure, urine output, pulse oximetry, and respiratory rate. If necessary, blood gases were analyzed but not routinely. All procedures were performed under intravenous anticoagulation using intravenous heparin with an initial bolus of 50–100 IU/kg followed by a 1000 IU/h perfusion. The maintenance dose was titrated to maintain the activated clotting time >300 s. Intracardiac catheters were inserted via the right and left femoral veins and included a 6F decapolar catheter placed into the coronary sinus, a 6F quadripolar catheter placed into the right ventricular apex, a standard linear multipolar or a high-definition mapping catheter (LiveWire 2-2-2 or Advisor HD Grid SE, Abbott Medical, Abbott Park, IL, USA), an ablation catheter, and an intracardiac echo probe (ViewFlex, Abbott Medical). Invasive blood pressure was monitored from the right femoral artery. The endo-epicardial geometries, substrates, and activation maps were generated using the EnSite Precision^TM^ 3D- EAM system (Abbott Medical). VT induction was performed with programmed electrical stimulation with up to three drive cycle lengths of 600 ms, 500 ms, and 400 ms and up to 3 extrastimuli from the right ventricular apex. If VT was not induced at baseline, isoprenaline was infused but not routinely. Bipolar voltage mapping was performed using <0.5 mV for dense scar and <1.5 mV for border zone. LAVAs were defined as sharp high-frequency ventricular potentials occurring anytime from the ventricular EGM [6,7,8,9]. LPs were defined as any low voltage EGM (<1.5 mV) with a single component or multiple continuous delayed components recorded after the surface QRS [6,7,8,9]. LAVAs and LPs were both annotated in the substrate map (Figure 2). Radiofrequency (RF) was delivered using a 3.5 mm open irrigated tip ablation catheter (FlexAbility SE, Abbott Medical). A deflectable sheath (Agilis NxT, Abbott Medical) was used in the case of transseptal access to the LV. In cases of epicardial mapping was required, the pericardium was accessed percutaneously using the method described by Sosa et al. before systemic heparinization. A steerable epicardial sheath (Agilis EPI, Abbott Medical) was used to facilitate epicardial navigation in epicardial procedures. The energy setting was 40–50 w, 43 °C maximum temperature, and 17 mL/min ablation catheter flow rate. After RF delivery, a final remap using the mapping catheter (LiveWire or HD Grid) was performed to evaluate substrate modification. Successful RF ablation was defined as the complete elimination of all LPs and LAVAs and the inability to induce VTs with programmed stimulation. In order to assess arrhythmia inducibility, programmed ventricular stimulation was repeated up to triple extrastimulus down to 200 ms or the ventricular refractory period.

### 2.5. Post-Ablation Management and Follow-Up

Patients visited the outpatient clinic regularly at 1, 6, and 12 months or whenever symptoms occurred after ablation. All patients underwent a 12-lead ECG and device (ICD or CRT-D) check during every visit.

### 2.6. Statistical Analysis

Continuous variables are expressed as mean ± standard deviation for normally distributed data or as median and inter-quartile range for skewed data and compared by Student’s *t*-test or the Mann–Whitney U test. Categorical variables were summarized as a percentage of the total compared by either the chi-square test or Fisher’s exact test. The statistical significance for all tests was accepted at *p* < 0.05. Kaplan–Meier analysis with log-rank test was used to calculate VT recurrence-free survival over time. Statistical analysis was performed using the SPSS v25 software (IBM SPSS Statistics).

## 3. Results

### 3.1. Patient Characteristics

Baseline clinical characteristics are summarized in Table 1.

The mean age was 67.56 ± 8.96 years, and 38 patients (74.5%) were men. Of the 51 patients, 68.7% had an endocardial-only ablation, and 31.3% had a combined endocardial and epicardial ablation. The two ablation groups were well-balanced in terms of baseline demographics. No significant differences in age, sex, LVEF, LVEDV, and significant cardiovascular comorbidities were found between groups. However, these differences among groups do not have any clinical correlation with the primary and secondary endpoints of the study.

### 3.2. Procedural Characteristics

The procedural results are shown in Table 2.

A mean of 2581.37 ± 2661.62 points was obtained during complete chamber mapping in sinus rhythm, with a mean of 831.33 ± 865.12 points when mapping was performed during VT. Significantly more points were acquired in the HD grid catheter group both in sinus rhythm and during VT (SR total points: 2060.78 ± 1600.38 vs. 3278.63 ± 3214.45, *p* = 0.05. VT total points: 4201.13 ± 5141.61 vs. 10,569.43 ± 13,644.94, *p* = 0.02). The overall procedural time was 204.93 ± 63.72, and the procedures were significantly longer when using the HD grid catheter (185.26 ± 36.61 vs. 220.5 ± 76.12, *p* = 0.027). Mapping time in sinus rhythm was also longer in the HD grid group (57.05 ± 27.72 vs. 89.87 ± 54.17, *p* = 0.02). Of note, the dimensions of the scar area, both bipolar and unipolar, were smaller in the HD grid group (Bipolar scar area, cm^2^: 4.52 ± 2.72 vs. 2.89 ± 2.81, *p* = 0.05. Unipolar scar area, cm^2^: 7.47 ± 4.55 vs. 5.56 ± 2.79, *p* = 0.03). Radiofrequency time was shorter in the group of HD grid mapping (30.52 ± 13.94 vs. 22.16 ± 11.03, *p* = 0.014). Interestingly, after radiofrequency delivery, at remap, significantly more LAVAs, and LPs were seen using the HD grid mapping catheter (LAVAs abolition: 99.47 ± 2.29 vs. 90 ± 21.06, *p* ≤ 0.001. LPs abolition: 99.64 ± 1.52 vs. 88.31 ± 27.09, *p* ≤ 0.001).

The HD Grid catheter was maneuvered throughout the LV endocardium retrogradely or via a transseptal access using a steerable sheath without difficulty. There were no cases of catheter entrapment within the mitral valve and sub-valvular apparatus. In severely dilated LV, we observed more issues obtaining a satisfying contact in the LV’s anterior/anterolateral basal segments when accessing the LV retrogradely, mainly because of the only curve available (D-F). Transseptal access has been performed to overcome this limitation. Another trick used in such cases was pushing the catheter, obtaining a large prolapse of the HD Grid catheter into the LV. During epicardial mapping, all regions of the LV and RV were accessible with the aid of a deflectable sheath. The complication rate was 9.8%, mainly due to vascular access (5.9%). There was one case of periprocedural transient ischemic attack and one case of pericardial effusion not requiring surgery (Table 3).

**Figure 3:**
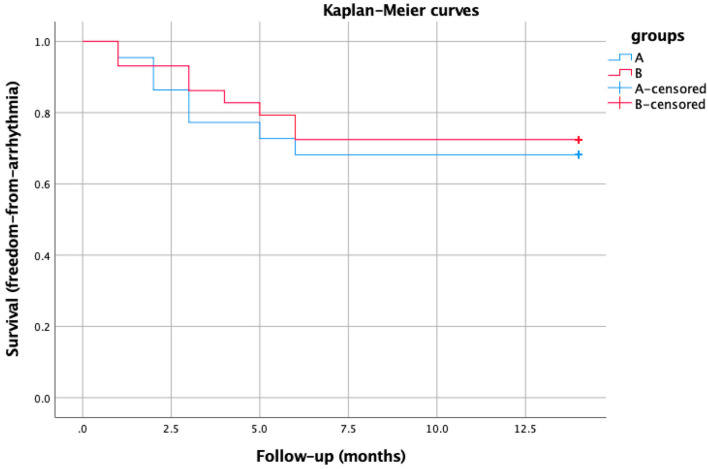
Kaplan–Meier survival curves showing no significant difference in the overall arrhythmia-free survival between the two groups.

We had no significant difference among the two groups regarding arrhythmia recurrences (group A 7 patients and group B 8 patients, 31.8% vs. 27.5%, *p* = ns). During a mean follow-up was 13.4 ± 3.6 months, we observed three deaths. During follow-up, two patients died because of worsening HF. Another patient with end-stage renal failure died because of sepsis after six months. Table 4 reports the cumulative follow-up in our study population. The overall arrhythmia recurrence rate was 29.4%. Among the recurrences were five slow VTs (monitor zone, no ICD intervention), four VTs requiring ATP, and six VT/VF requiring ICD shock. Nine patients were admitted because of an arrhythmic recurrence, while eight experienced worsening of the underlying HF. Three patients underwent a redo VT ablation during the follow-up period. Kaplan–Meier analysis showed no significant difference in the overall arrhythmia-free survival between the two groups (log-rank, *p* = 0.329) (Figure 3).

## 4. Discussion

In this prospective study comparing two different mapping catheters in patients undergoing VT ablation, we showed the clinical usefulness of a novel grid mapping catheter to guide VT ablation. The HD grid mapping catheter was feasible and safe to maneuver for both endocardial and epicardial mapping during sinus rhythm and VT. In our population, this high-definition multipolar grid mapping catheter was associated with significantly shorter radiofrequency delivery time and mapping time during VT. Conversely, we had significantly longer procedural and mapping time in sinus rhythm. In addition, significantly more total points were acquired in sinus rhythm and during VT. However, VT’s used points were not statistically different, while a trend was observed for the points obtained and utilized in sinus rhythm. A possible explanation that would justify the longer procedural time may be the greater amount of information collected by the mapping catheter and the more elaborate post-processing analysis of the acquired points. This would also be justified by the longer duration of the mapping during sinus rhythm. However, mapping during tachycardia was significantly shorter due to the grid mapping catheter’s specific design and higher definition.

Multielectrode mapping for ventricular tachycardia has been proven to be safe and effective and better discriminate LPs due to a lower sensibility of far-field signals [10,11].

Interestingly, the low voltage areas (bipolar and unipolar dense scar) detected using the HD grid were smaller than the linear duodecapolar mapping catheter due to the higher substrate definition given by this mapping catheter. Recently, Jiang et al. reported similar findings regarding a higher definition of the scar area and a smaller extension of the scar by using the HD grid catheter. The authors also demonstrated that the automated activation mapping with the HD grid catheter enabled the rapid acquisition of high-definition maps to display the VT circuit’s critical components, successfully targeted for ablation [12]. The authors also showed that local EGM properties varied between the two orthogonal bipolar directions (along and across the splines). Takigawa et al. observed that the bipolar voltage and distribution of LAVAs may differ significantly between diagonally orthogonal bipolar pairs [13]. They observed a median variation of bipolar voltage of 0.28 mV and a LAVAs missing rate of 30% between diagonally orthogonal bipole pairs. Although the advantages given by the HD grid catheter’s orthogonal bipolar designs, there may be a downside related to larger inter-electrode spacing. The same authors’ group demonstrated that an electrode pair with larger spacing was more likely to sense higher far-field voltages, and some LAVAs could be mistaken or missed. Differently, closer electrode spacing is superior for identifying surviving tissue in the scar [14]. As previously described, the recorded signals are affected by contact, electrode size, interelectrode distance, angle of contact, and the relationship of the electrodes to the activation wavefront. The resolution of mapping when using standard catheters is poor and often misleading. Small electrode catheters with closer interelectrode distance are becoming the cornerstone of any approach to the atrial and ventricular substrate to understand the underlying substrate better. The smaller the electrode and the interelectrode space, the more closely the signal approximates the filtered unipolar signal, which is the accurate local activation.

Moreover, the multielectrode and high-definition mapping may also allow rapid mapping of VTs to correlate substrate and function better. Finally, we observed more LAVAs and LPs during remapping with the HD grid after radiofrequency delivery, requiring further ablation and prolonging procedural time. Since there was no difference among the two groups in terms of arrhythmia-free survival at follow-up, we could postulate whether such a high-definition of the substrate could increase the efficacy and safety of the procedure. These factors need to be further evaluated in larger studies.

## 5. Study Limitations

The study has several limitations. First, it was not a randomized study, and a direct mapping comparison in the same procedure among the two catheters was not performed. However, we compared the HD Grid mapping catheter with the linear duo-decapolar catheter, which was previously validated and served as our standard-case mapping catheter. The follow-up duration was limited to slightly more than 12 months, although early results appeared promising. Finally, the study’s primary endpoints were the total RF delivery time, the number of points acquired in sinus rhythm and VT, and the scar area. Although we recognize that more robust clinical endpoints are of interest, such as long-term freedom from arrhythmia recurrences, the study was not designed to prove this concept.

## 6. Conclusions

VT mapping and ablation guided by the HD Grid mapping catheter is safe and feasible in VT ablation via both endocardial and epicardial approaches. Recordings from a grid-shaped catheter allow the operators to acquire significantly more information regarding the substrate. Given this abundance of data, an accurate signal analysis on- and offline is necessary to identify the mapping target amenable of interest and effectively guide the ablation procedure.

## Figures and Tables

**Table 1:** CVD = cardiovascular disease, COPD = chronic obstructive pulmonary disease, ICD = implantable cardioverter defibrillator, CRT-D = cardiac resynchronization therapy–defibrillator, HF = heart failure, ICM = ischemic cardiomyopathy, NICM = non-ischemic cardiomyopathy, LVEF = left ventricular ejection fraction, LVEDV = left ventricular end-diastolic volume, BB = beta-blockers, ACEi = angiotensin-converting enzyme inhibitors, ARBs = angiotensin II receptors blockers, MRAs = mineralocorticoid receptors antagonists, OAT = oral anticoagulation therapy.

	Total (*n* = 51)	Group A (*n* = 22)	Group B (*n* = 29)
**Males, *n* (%)**	38 (74.5%)	15 (68.1%)	23 (79.3%)
**Age, mean ± SD**	67.56 ± 8.96	68.3 ± 6.6	66.9 ± 10.5
**Diabetes, *n* (%)**	15 (29.4 %)	6 (27.2%)	9 (31%)
**Hypertension, *n* (%)**	34 (66.6 %)	16 (72.7%)	18 (62%)
**Family Hx of CVD, *n* (%)**	19 (37.2 %)	8 (36.3.9%)	11 (37.9%)
**Smoke, *n* (%)**	28 (54.9 %)	11 (50%)	14 (48.2%)
**Dyslipidemia, *n* (%)**	29 (56.8%)	13 (59.1%)	16 (55.1%)
**Obesity, *n* (%)**	15 (29.4%)	7 (31.8%)	8 (27.5%)
**COPD, *n* (%)**	12 (23.5%)	5 (22.7%)	7 (24.1%)
**Renal failure, *n* (%)**	19 (37.2%)	9 (40.9%)	10 (34.4%)
**Creatinine (mg/dL), mean ± SD**	1.22 ± 0.40	1.25 ± 0.45	1.20 ± 0.37
**ICD, *n* (%)**	37 (72.5%)	17 (77.2%)	20 (68.9%)
**CRT-D, *n* (%)**	14 (27.4%)	6 (27.2%)	8 (27.5%)
**NYHA I, *n* (%)**	9 (17.6%)	4 (18.8%)	5 (17.2%)
**NYHA II, *n* (%)**	30 (58.8%)	13 (59.1%)	17 (58.6%)
**NYHA III, *n* (%)**	9 (17.6%)	4 (18.8%)	5 (17.2%)
**NYHA IV, *n* (%)**	3 (5.8%)	1 (4.5%)	2 (6.8%)
**HF hospitalization last 6 m, *n* (%)**	19 (37.2%)	11 (50%)	8 (27.5%)
**ICM, *n* (%)**	34 (66.6%)	15 (68.1%)	19 (65.5%)
**NICM, *n* (%)**	13 (25.4%)	6 (27.2%)	8 (27.5%)
**Myocarditis, *n* (%)**	4 (7.8%)	1 (4.5%)	2 (6.8%)
**Previous ablation, *n* (%)**	8 (15.6%)	3 (13.6%)	5 (17.2%)
**Atrial fibrillation, *n* (%)**	16 (31.3%)	7 (31.8%)	9 (31%)
**Atrial tachycardia, *n* (%)**	2 (3.9%)	1 (4.5%)	1 (3.4%)
**Atrial flutter, *n* (%)**	6 (11.7%)	3 (13.6%)	3 (10.3%)
**LVEF, mean ± SD**	29.44 ± 9.21	31.52 ± 9.41	27.79 ± 8.89
**LVEDV, mean ± SD**	191 ± 54.11	189.68 ± 58.74	192.29 ± 51.40
**Drugs**			
- **Amiodarone, *n* (%)**	38 (74.5%)	16 (72.7%)	22 (75.8%)
- **BB, *n* (%)**	48 (94.1%)	20 (90.9%)	28 (96.5%)
- **Mexiletine, *n* (%)**	11 (21.5%)	5 (22.7%)	6 (20.6%)
- **ACEi/ARBs, *n* (%)**	39 (76.4%)	18 (81.8%)	21 (72.4%)
- **MRAs, *n* (%)**	24 (47%)	9 (40.9%)	15 (51.7%)
- **Sacubitril, *n* (%)**	10 (19.6%)	4 (18.1%)	6 (20.6%)
- **Diuretics, *n* (%)**	41 (80.3%)	18 (81.8%)	23 (79.3%)
- **Antiplatelet, *n* (%)**	35 (68.6%)	17 (77.2%)	18 (62%)
- **OAT, *n* (%)**	20 (39.2%)	9 (40.9%)	11 (37.9%)

**Table 2:** VT = ventricular tachycardia, LAVAs = local abnormal ventricular activities, LPs = late potentials.

	Total (*n* = 51)	Group A (*n* = 22)	Group B (*n* = 29)	*p*
**Radiofrequency time, min ± SD**	25.86 ± 12.94	30.52 ± 13.94	22.16 ± 11.03	0.014
**Procedure time, min ± SD**	204.93 ± 63.72	185.26 ± 36.61	220.5 ± 76.12	0.027
**Fluoroscopy time, min ± SD**	23.32 ± 11.56	25.1 ± 11.82	22.1 ± 11.42	0.34
**Sinus Rhythm points, *n* ± SD**	2581.37 ± 2661.62	2060.78 ± 1600.38	3278.63 ± 3214.45	0.05
**Sinus Rhythm total points, *n* ± SD**	14,908.97 ± 11,161.37	11,925.06 ± 10,932.56	16,788.47 ± 11,340.50	0.04
**VT points, *n* ± SD**	831.33 ± 865.12	850.88 ± 959.74	809 ± 819.14	0.87
**VT total points, *n* ± SD**	7173 ± 10,189.51	4201.13 ± 5141.61	10,569.43 ± 13,644.94	0.02
**Map time Sinus Rhythm, min ± SD**	71.52 ± 44.01	57.05 ± 27.72	89.87 ± 54.17	0.02
**Map time VT, min ± SD**	20.81 ± 13.50	23.70 ± 13.44	13.57 ± 7.45	0.11
**VT cycle length (ms), mean ± SD**	374 ± 73.01	377.27 ± 83.28	372.71 ± 68.2	0.63
**Number of VT ablated, mean ± SD**	1.13 ± 1.11	1.12 ± 1.35	1.08 ± 0.88	0.12
**LAVAs area, cm^2^ ± SD**	0.31 ± 0.21	0.51 ± 0.63	0.16 ± 0.33	0.02
**LPs area, cm^2^ ± SD**	0.74 ± 0.90	0.86 ± 0.78	0.66 ± 0.98	0.80
**N. of LV segments with LPs, *n* ± SD**	2.65 ± 1.73	3.05 ± 1.74	2.33 ± 1.68	0.69
**Abolition of LAVAs (%), mean ± SD**	94.18 ± 16.36	99.47 ± 2.29	90 ± 21.06	<0.001
**Abolition of LPs (%), mean ± SD**	93.27 ± 20.79	99.64 ± 1.52	88.31 ± 21.09	<0.001
**Bipolar scar area, cm^2^ ± SD**	3.67 ± 2.86	4.52 ± 2.72	2.89 ± 2.81	0.05
**Unipolar scar area, cm^2^ ± SD**	6.47 ± 3.81	7.47 ± 4.55	5.56 ± 2.79	0.03

**Table 3:** Periprocedural complications.

**Total complications, *n* (%)**	5 (9.8%)
**Pericardial tamponade, *n* (%)**	1 (1.9%)
**Groin hematoma/AV fistula, *n* (%)**	3 (5.9%)
**Intraprocedural death, *n* (%)**	0 (0%)
**Transient ischemic attack (TIA), *n* (%)**	1 (1.9%)

**Table 4:** Cumulative follow-up.

**Total VT/VF, *n* (%)**	15/51 (29.4%)
- **VT in monitor zone, *n* (%)**	5/15 (33.3%)
- **VT treated with ATP, *n* (%)**	4/15 (26.6%)
- **VT/VF treated with shock, *n* (%)**	6/15 (40%)
**Arrhythmic storm, *n* (%)**	1/51 (1.9%)
**VT/VF requiring hospitalization, *n* (%)**	9/51 (17.6%)
**HF hospitalization, *n* (%)**	8/51 (15.6%)
**AF, *n* (%)**	9/51 (17.6%)
**Death, *n* (%)**	3/51 (5.8%)
**Arrhythmic death, *n* (%)**	1/51 (1.9%)

## Data Availability

Data are stored in the Hospital secured database.
